# Carbapenemase-Producing Enterobacteriaceae detected in a Large Canadian Tertiary Care Hospital: Five-year retrospective study

**DOI:** 10.1017/ash.2024.338

**Published:** 2024-09-16

**Authors:** Ashley James, Melisa Avaness, Victoria Williams, Lorraine Maze dit Mieusement, Heather Candon, Jerome Leis

**Affiliations:** Sunnybrook Health Science Center; University of Toronto

## Abstract

**Background:** The prevalence of carbapenemase-producing Enterobacteriaceae (CPE) is increasing worldwide. In Canada, where rates of healthcare-associated (HA) transmission of CPE remains relatively low, there is a need to share early experience of universal screening programs and risk factors for HA acquisition. **Method:** In 2018, universal screening was introduced throughout our large Canadian tertiary care hospital across, all critical care and oncology units. Additionally, risk-factor based screening was applied in all other inpatient units, with further targeted screening of roommate exposures or all inpatients on unit following identification of a single HA case. A retrospective cohort study was carried out on CPE cases detected between January 2018 and December 2023. We assessed the proportion of HA CPE cases, defined as CPE identified in patients with prior admission to our facility or after >72 hours after admission. HA cases were examined for relevant risk factors, including known roommate with CPE, the presence of other CPE on the unit, exposure to outbreak units, prior travel history, travel by a family member, and antibiotic exposure within the past 90 days. **Result:** A total of 150 CPE cases were identified, with 66 (44%) classified as HA. Among these HA cases, 14 (21%) were associated with presence of known case on the unit. The remaining 52 (79%) represented sporadic nosocomial cases without a known exposure or further transmission on the unit. Upon further retrospective review, 6 (9.2%) HA cases had documented travel history or exposure to a family member with recent travel to China, India, Sri Lanka, or the United States within the past year. Nearly all HA cases (62, 95.4%) had antibiotic exposure within 90 days of CPE detection; specifically, 47 (72.3%) received beta-lactams, 42 (64.6%) cephalosporin, 25 (38.5%) glycopeptide, 20 (30.8%) carbapenem, and 8 (12.3%) macrolide. **Conclusion:** HA CPE acquisition identified during the first 5-years of universal screening were mostly sporadic and not associated with known exposures or other risk factors. Receipt of prior antibiotics was present in nearly all cases.

